# Multi-scale visual analysis of time-varying electrocorticography data via clustering of brain regions

**DOI:** 10.1186/s12859-017-1633-9

**Published:** 2017-06-06

**Authors:** Sugeerth Murugesan, Kristofer Bouchard, Edward Chang, Max Dougherty, Bernd Hamann, Gunther H. Weber

**Affiliations:** 1 Computational Research Division, Lawrence Berkeley National Laboratory, One Cyclotron Road, Berkeley, 94720 CA USA; 2 Department of Neurological Surgery, UCSF, 505 Parnassus Ave, San Francisco, 94143 CA USA; 3 Department of Computer Science, University of California, One Shields Avenue, Davis, 95616 CA USA

**Keywords:** Electrocorticography, Clustering, Spatio-temporal graphs, Unsupervised learning, Neuroinformatics, Epilepsy, Visual analysis, Brain imaging, Graph visualization, Mutli-scale analysis

## Abstract

**Background:**

There exists a need for effective and easy-to-use software tools supporting the analysis of complex Electrocorticography (ECoG) data. Understanding how epileptic seizures develop or identifying diagnostic indicators for neurological diseases require the in-depth analysis of neural activity data from ECoG. Such data is multi-scale and is of high spatio-temporal resolution. Comprehensive analysis of this data should be supported by interactive visual analysis methods that allow a scientist to understand functional patterns at varying levels of granularity and comprehend its time-varying behavior.

**Results:**

We introduce a novel multi-scale visual analysis system, ECoG ClusterFlow, for the detailed exploration of ECoG data. Our system detects and visualizes dynamic high-level structures, such as communities, derived from the time-varying connectivity network. The system supports two major views: 1) an overview summarizing the evolution of clusters over time and 2) an electrode view using hierarchical glyph-based design to visualize the propagation of clusters in their spatial, anatomical context. We present case studies that were performed in collaboration with neuroscientists and neurosurgeons using simulated and recorded epileptic seizure data to demonstrate our system’s effectiveness.

**Conclusion:**

ECoG ClusterFlow supports the comparison of spatio-temporal patterns for specific time intervals and allows a user to utilize various clustering algorithms. Neuroscientists can identify the site of seizure genesis and its spatial progression during various the stages of a seizure. Our system serves as a fast and powerful means for the generation of preliminary hypotheses that can be used as a basis for subsequent application of rigorous statistical methods, with the ultimate goal being the clinical treatment of epileptogenic zones.

**Electronic supplementary material:**

The online version of this article (doi:10.1186/s12859-017-1633-9) contains supplementary material, which is available to authorized users.

## Background

The human brain is a highly connected, dynamic system comprised of specialized brain regions that coordinate and interact in many complex ways for communication, producing intricate patterns of system behavior [1]. Analyzing these communication patterns can help us gain an understanding of the normal functioning of the brain, how we learn or age, and how neurological disorders develop or can be treated [1, 2]. Brain systems function across a large range of spatial and temporal scales. Investigating how the connectivity patterns vary across these different scales has provided new insights into how low-level signals cause global brain state transformations [3]. To support such analysis and capture these patterns comprehensively, data with high temporal and spatial resolution and the low signal-to-noise ratio is needed.

Recent advances in invasive monitoring technologies such as electrocorticography (ECoG) have risen to this challenge by recording high-resolution electrical signals captured by electrodes placed directly on the cortical surface of the brain. The correlation of electrical activity between these electrodes yields a measure of functional connectivity between them. As the derived functional network changes over time, the topology and the attributes of the network vary as well, making it difficult to analyze and visualize the network.

Developments in graph theoretical methods have made it possible to simplify and characterize the data contained in the connectivity network. For example, through community detection methods, it has been determined that brain networks exhibit modular organization [4], i.e., they consist of clusters—subsets of regions having strong inter-modular connections and sparse inter-modular connections. These clusters represent specialized behavioral systems such as higher-order vision, or sensory-motor processing [5].

One way to explore how these behavioral systems interact when performing a task or are impaired due to neurological disorders is to study how the modules evolve over time [2]. This study involves identifying cluster evolution patterns such as: spatial distribution, or a combination of clusters; electrical activation or deactivation of a cluster; and the birth and death of clusters. In the case of epilepsy, for instance, visual analysis of the cluster data combined with the electrical activity can help differentiate normal and ictal (seizure) states of the brain. These patterns—when validated with statistical analysis—are crucial for a successful treatment of the identified epileptogenic zones.

The spatio-temporal patterns in time-varying clusters appear at different spatial and temporal scales. To capture and analyze these patterns, it is important that the temporal scale of the analysis matches the temporal scale of the patterns themselves [6]. For example, patterns such as spatial distribution or combinations of clusters are best captured at a finer temporal scale while global transitions of brain states are captured at a coarser temporal scale. Analyzing the patterns at varying granularity is crucial as appropriate scales for evaluation are not obvious a priori and a single optimal solution at a particular scale is unlikely to exist [6].

Existing approaches to visualize dynamic spatio-temporal clusters operate mostly at a single temporal scale and do not satisfactorily support the in-depth comparison and evaluation of the evolution patterns underlying the data. They mainly focus on visualizing such data by directly depicting all of the information through visual representations or using computational methods to reduce and summarize the visual data. While direct depiction methods suffer from scalability issues, data reduction methods ignore the low-level details of the dataset that are important in explaining high-level evolution patterns.

**Fig. 1:**
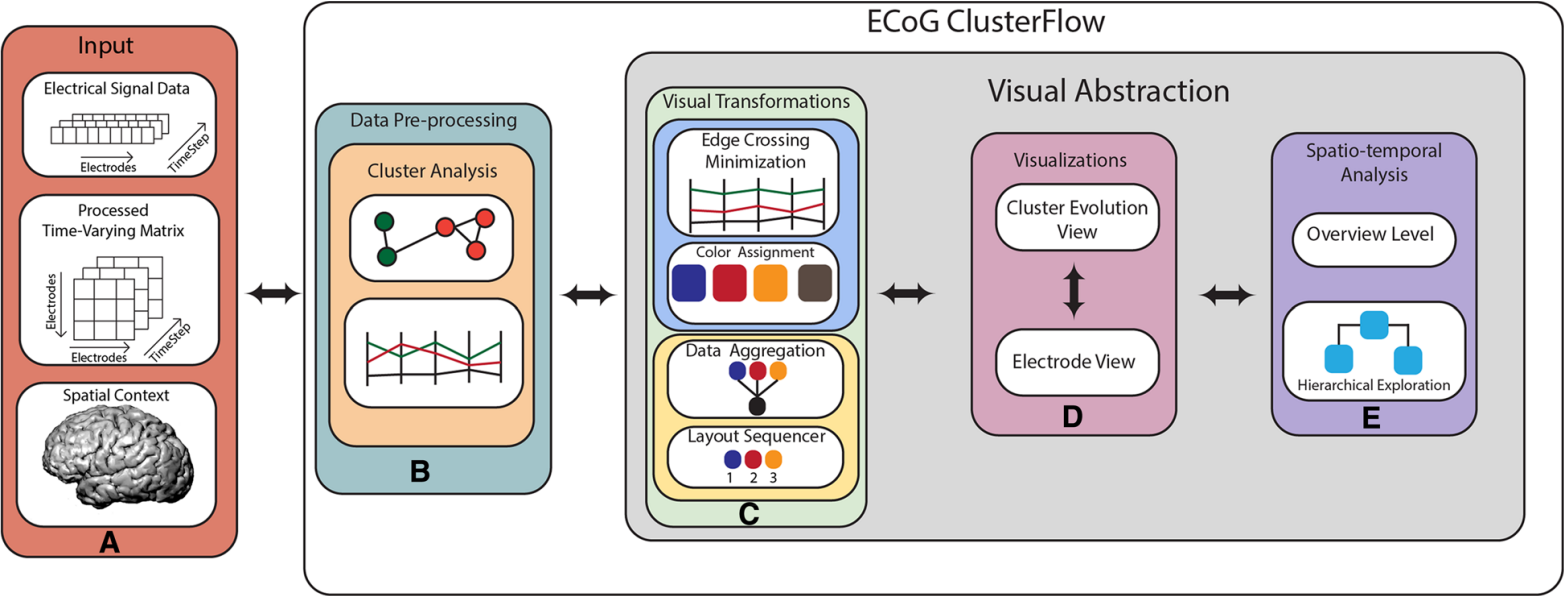
Overview of the ECoG ClusterFlow pipeline. **a** Raw electrical signals are statistically analyzed to derive the dynamic network data. **b** The data pre-processing step identifies and links cluster across timesteps. **c** Main modules of the visualization system. **d** Users can investigate patterns in two major visualization views. **e** Users can perform various types of spatio-temporal analysis based on these views

**Fig. 2:**
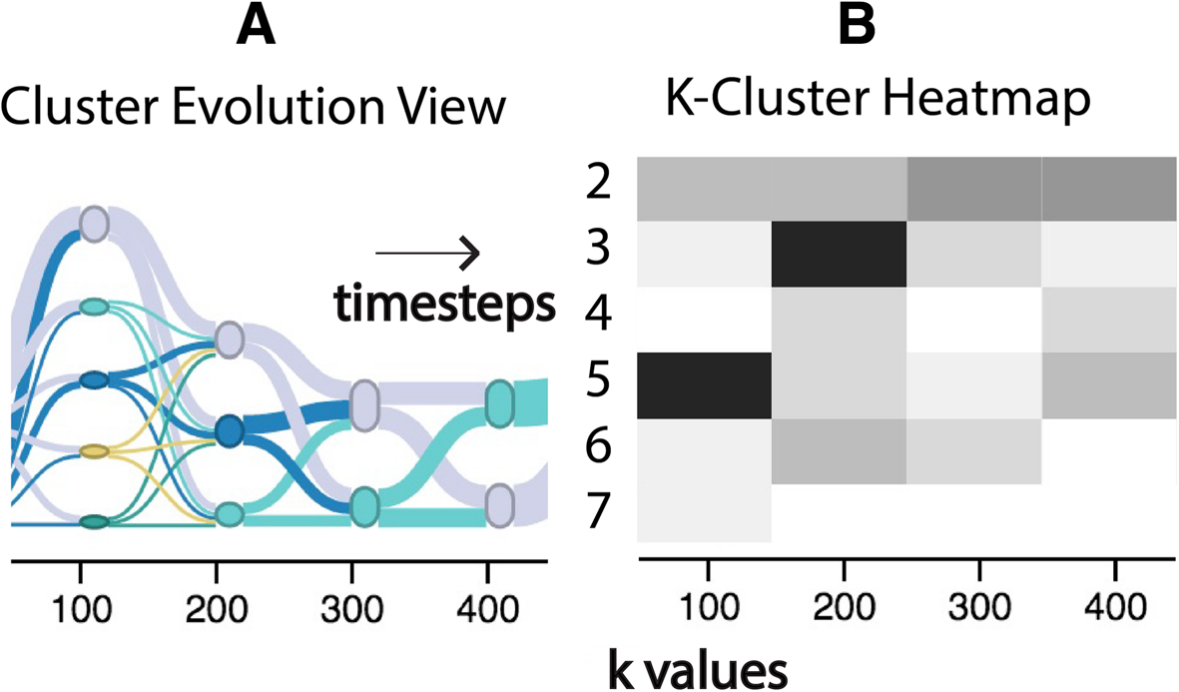
Evolution of clusters for four timesteps. **a** The cluster evolution view shows clusters and transitions between them. The nodes have colors based on their cluster membership. **b** The K-cluster heatmap on the bottom visualizes the likelihood of a range of K values that determine the final number of clusters for a particular timestep, for e.g. a clear maxima is evident for 100 ms (K as 5) and 200 ms (K as 3)

To support a comprehensive and detailed study of ECoG data, we present ECoG ClusterFlow (Fig. 1), an interactive system that supports the exploration, comparison and analysis of time-varying community evolution patterns at varying temporal granularity through two major views: 1) an overview (Fig. 2) summarizing the overall changes in cluster evolution, where users explore salient dynamic patterns; and 2) a hierarchical glyph-based timeline visualization for exploring the dynamic spatial organizational changes of the clusters that uses data aggregation [7] and small multiples [8] methods.

These techniques allow users to gain insights at many levels of temporal granularity, exploring globally evolving patterns to observing small-scale spatial changes. In summary, our main contributions include:

- A hierarchical multi-scale approach to visualizing temporal modular changes in brain networks
- Unique glyph-based designs that explore spatial organizational changes of the dynamic cluster configuration

Furthermore, the specific design goals and capabilities of our system were articulated in close collaboration with the neuroscientists and the neurosurgeon on our team, ensuring that our prototype improves the overall data exploration process. Our system was repeatedly evaluated and tested by the users, making possible the development of analysis modules that help gain new insights into the data. We present two case studies using synthetic and epileptic seizure datasets to demonstrate the usefulness of our system.

## Related work

Work related to ours falls into three categories: visualization of communities for dynamic graphs; visualization of spatio-temporal data; and visualization systems for studying brain connectivity in ECoG data.

### Communities for dynamic graphs

When exploring communities in dynamic graphs, existing techniques primarily use animation (time-to-time mapping) or static timeline-based (time-to-space mapping) visualization methods to depict modular changes over time.

In **animations**, the community structure of the network is shown by color-coding the nodes or partitioning the drawing space into sections [9–12] or nested blocks [13] (if the data is hierarchical). Due to their reliance on short-term memory, animations increase the cognitive load during analysis [14]. One way to mitigate this problem is to maintain the ‘mental map’ of the layout by minimizing node movement in the animation [15]. An alternate approach to decrease the cognitive load is to place multiple graph representations along a timeline using small multiples [16]. However, this multi-view approach leaves the user with the manual task of assimilating and identifying changes.

To address this problem, several approaches utilize **timeline-based representations** [17–19], visualizing only the evolution of clusters over time. In a timeline view, each segment along the axis perpendicular to the timeline represents a cluster identified at that particular timestep. The links between two axes represent the changes in the cluster affiliation of the nodes. The arbitrary ordering of the nodes along the vertical axis may increase link crossings between axes, inhibiting easy comprehension of the evolution patterns. To address this issue, Reda et al. [18] and Sallabury et al. [20] employ sorting techniques to place active and stable communities at the top of the vertical axis.

To further support the comprehension of transitions between communities, alluvial diagrams [21] model the links between clusters in different vertical axes as split-merge ribbons [17, 22, 23]. This approach enhances the visual traceability of important cluster evolution patterns.

Reda et al. [24] visualize the evolution of time-varying clusters while taking into account the spatial context, and by linking a space-time cube with a timeline representation. In contrast, our method provides the spatial context showing a multi-scale dynamic evolution patterns in 2D space, reducing visual clutter and occlusion. Our technique matches clusters through a best overall match algorithm, enabling intuitive identification of time-varying community patterns.

### Spatio-temporal data

Previous visual analysis methods for spatio-temporal data utilize either integrated or separated views [25].

**Integrated views** visualize spatial and temporal data in one view. Superimposing temporal graph data onto a spatial view [26] and visualizing a 3D space-time cube timeline over a 2D spatial view [27] are two examples of integrated views. Another hybrid 2.5D approach proposed by Tominski et al. [28] displays temporal information on top of a 2D spatial layout. However, for a large number of timesteps or data points, these views can easily become cluttered and occluded.

**Separated views** overcome visual clutter by using dedicated views to present different aspects of spatio-temporal data. Plug et al. [29] link data in spatial and temporal domains by using small multiples of maps, superimposing a subset of temporal data on each of the spatial maps. Jern et al. [30] utilize color to link spatial and temporal data. Other methods [31] for static data use interaction techniques to link data in both domains, requiring substantial and concentrated eye movements for visual analysis.

To overcome such drawbacks, visual glyph designs aggregate spatio-temporal attributes that not only reduce the size of the represented data but also enable intuitive comparison of temporal data. *Glidgets* [32] depict temporal changes by segmenting glyphs into time slices, enabling the comparison of attributes over time. Related work by Nan Cao et al. [33] and Erbacher et al. [34] uses glyphs that aggregate temporal data to summarize the entire dataset with the overall goal of detecting anomalous behavior in the network.

ECoG ClusterFlow uses a combination of the aforementioned concepts to provide unique glyph-based designs and visual analysis methods that show the overall modular changes of the network.

### Visualization systems for ECOG brain connectivity data

Graimann et al. [35] presented methods to visualize event-related desynchronization and synchronization (ERD/ERS) patterns of implanted electrodes. Research done by Korzeniewska et al. [36] and Cristhian et al. [37] included the visualization of causal relationships among electrode sites. Kubanek et al. [38] recently presented a tool for visualizing topographies of ECoG cortical activity on a 3D model of the cortex. Although these approaches satisfactorily portray the spatial layout of the brain, they do not support the visualization of time-varying modular data for functional ECoG brain networks.

There exists a need for tools supporting efficient, high-level data analysis and exploration, including dynamic cluster analysis as a main focus. To aid the process of generating and verifying scientific hypotheses, a thorough visual understanding of the intricate spatio-temporal patterns of ECoG data is necessary. We address this need with a stand-alone application that allows a user to explore cluster community evolution at varying granularity.

## Cluster detection

Our visualization methods are based on sequence of communities detected at each timestep. We call this sequence of communities **dynamic communities** or **dynamic clusters**. Given the graph at a particular timestep *G*={*N,E*}, where *N* are the nodes that represent electrodes and *E* are the edges that represents the correlation between the electrodes, the community detection algorithm clusters the data into K non-overlapping and exhaustive communities.

Derivation of time-dependent clusters is an essential task in the analysis of time-varying brain network [39]. Two main approaches [40] are commonly used: 1) A two-stage approach derives communities at each timestep and then tracks them over time using different community tracking methods [20, 41]. 2) An evolutionary clustering approach takes into account the graph topology and the clustering results from previous timesteps. Based on the feedback from the neuroscientists on our team and other existing work [20, 39], we choose the two-stage clustering approach (described in detail in “Cluster tracking” Section) with consensus clustering [42] as our primary detection algorithm. This method produces a better quality of clustering results since each timestep is clustered locally (determining the statistically correct number of clusters) [20], and combines the best outputs of multiple runs of the K-means clustering algorithm.

## Methods

We developed ECoG ClusterFlow in close collaboration with neuroscientists (including neurosurgeons) to guide the design of our analysis framework and to ensure that it would be truly valuable as an exploratory tool.

Figure 1 shows the pipeline of our system. The input to our system is: 1) the processed electrical signal data originating from each electrode in the ECoG grid and, 2) its corresponding pairwise dynamic correlation network. The dynamic network data is pre-processed to derive dynamic clusters. Visualization methods, such as data aggregation, are applied to the cluster data in the pre-computation phase and final visualizations are generated.

Based on our conversations with domain experts and the network task taxonomy by Ahn et al. [43], we have identified the following domain questions of interest:

- **Identify temporal brain states (Q1):** What activation patterns are consistent over a continuous period of time?
- **Identify transitions between brain states (Q2):** Given the brain states, what patterns characterize their transition to another state?
- **Compare the evolution patterns associated with different brain states (Q3):** What patterns underlie the brain states during normal versus diseased condition?
- **Assess changes in community membership (Q4):** Given a spatial region of interest in the brain, how do the clusters belonging to these regions change over time?

These questions led us to establish the following system design goals:

- **Timeline-based visualizations (G1):** Support views that display the time-varying cluster information on a static display to take advantage of the user’s visual perception instead of cognition (time-to-time mapping)
- **Multiple levels of detail and abstraction (G2):** Support views that enable neuroscientists to explore the data at multiple levels of granularity for analysis
- **Holistic visualizations (G3):** Support visual designs that combine multiple data attributes like cluster membership and its electrical activation

These goals are addressed in our system by two major views: the *Cluster Evolution View* and the *Electrode View*.

### Cluster evolution view

**Fig. 3:**
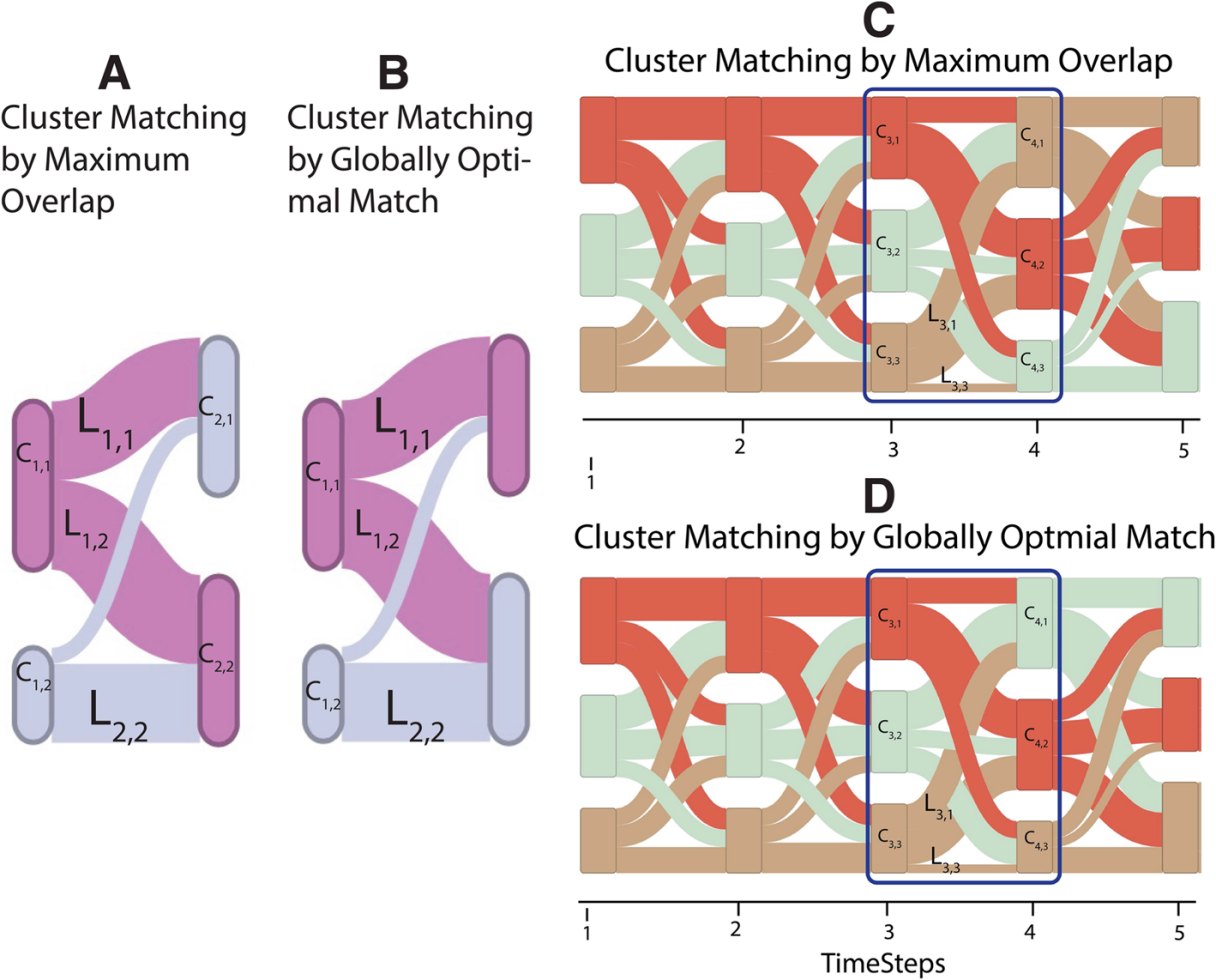
Comparison of tracking algorithms in artificial datasets. In image **c** the maximum overlap algorithm pairs *C*_3,3_ to *C*_4,1_, while in **d** the globally optimal matching algorithm pairs *C*_3,3_ to *C*_4,3_, qualitatively making communities more visually traceable in image **d**. The scalar values for the links in image A and B are *L*_1,1_=11, *L*_1,2_=11, *L*_2,1_=10, *L*_2,2*s*_=2

The cluster evolution view (Fig. 3) highlights the salient patterns of the cluster evolution including the emergence, death, contraction, expansion, merging and splitting of clusters (Q2, Q3, Q4). Through this view, analysts can compare and analyze modular signatures (cluster evolution patterns) over time and identify important time intervals and distinct brain states. The cluster evolution patterns are represented using a flow-based visualization [21, 22] (G1) (alluvial diagram), where the clusters metaphorically flow like a river with split/merge tributaries from left to right.

Formally, at each timestep *t* on the horizontal axis, rectangular blocks represent clusters *C*_*t,i*_ where the height of each block corresponds to the cluster’s size at that timestep. Flow-based transition links *L*_*i,j*_, where *i* is the source community and *j* is the sink community, connect clusters to show changes in the community structure over time. We model these links as Bezier curves, to generate a continuous representation of the transition between successive communities [22]. Figure 3 shows the evolution of dynamic clusters for five timesteps. Furthermore, to easily assess the community membership in dynamic clusters, we color communities using solid coloring, using N perceptually distinct colors from a qualitative colorbrewer [44] colormap.

#### Cluster tracking

To support the two-stage cluster detection approach, it is necessary to determine correspondences between clusters in consecutive timesteps. Based on the input from neuroscientists, we have investigated two approaches to compute this matching: (1) maximum overlap tracking and (2) computing the globally optimal match.

Maximum overlap tracking (Fig. 3 a, c) is a greedy algorithm that iteratively matches the two clusters in consecutive timesteps that share the maximum number of electrodes. This process is repeated until no overlapping clusters remain. This approach may not always produce an intuitive correspondence between clusters. For example, in Fig. 3 a and c, clusters *C*_1,2_ and *C*_2,2_ have maximum overlap (of 11 electrodes) and are paired in the first iteration. This only leaves *C*_2,1_ as possible match for *C*_1,2_ in the second iteration, even though the overlap between *C*_1,2_ and *C*_2,1_ is relatively small (only two electrodes).

To find the globally optimal assignment, our second approach picks the best overall match between all clusters in consecutive timesteps. We define a similarity measure

$$ sim = \frac{\left|C_{t,i} \cap C_{t+1,j}\right|}{\left|C_{t,i} \cup C_{t+1,j|}\right|} $$

between clusters *C*_*i*_ and *C*_*j*_ in consecutive timesteps *t* and *t+1*, similar to the approaches by Greene et al. [45] and Sallabury et al. [20]. Next, we compute a similarity matrix comprised of the pairwise similarity measures between all possible cluster combinations. To avoid matching of clusters with small overlap, we set to zero those similarity values that are below a threshold *θ*. To match clusters, we consider all possible cluster matchings between timesteps—by considering all possible permutations of clusters—and compute a global similarity value as the sum of the similarity values for all matched clusters. The overall best match is the permutation that maximizes global similarity. While considering all possible permutations is computationally expensive, we usually consider only a small number of clusters (approximately seven) per time step, keeping this approach tractable. Figure 3 b shows the best overall match for our example, matching clusters *C*_1,1_ and *C*_2,1_ as well as clusters *C*_1,2_ and *C*_2,2_, a more intuitive choice than the result obtained by maximum overlap tracking. Figure 3 shows an example of this approach for an artificial dataset with three dynamic communities. The two approaches differ in the community results starting at timestep four.

#### Sorting and ordering of nodes

To enhance the visual traceability of the clusters, the node layout of the graph should ideally minimize edge crossings with optimal ordering of the nodes (clusters) at each vertical axis. To determine such an ordering, we must take all the timesteps into consideration. Several methods have been proposed to compute such an ordering [20, 22]. Our approach handles more timesteps by not considering the individual elements contained within clusters and dividing the sorting procedure into N individual blocks of T timesteps. To reduce the computational complexity—to achieve the least start-up-time of 40–60 s and to scale to up to 60 timesteps—our heuristic solution (barycenter approach [46]) sweeps horizontally across all the clusters over N blocks of T timesteps (where NT is the total number of timesteps in the data) in a front-to-back and back-to-front manner to optimize the order and position of these communities on the vertical axis. This procedure results in a cluster ordering that minimizes the link distance between the timesteps.

#### K-Cluster heatmap

Cluster analysis results can be sensitive to noise and prone to overfitting [42]. The K-Cluster heat map produces important information for the evaluation of the uniqueness of the number of clusters detected per timestep. Consensus clustering uses a cumulative distribution function (CDF) to determine an appropriate number of clusters K. In most cases, the likelihood for a single value of K will be large compared to the others, and the confidence that the chosen number of clusters is correct is high. The K-Cluster heat map (Fig. 2) shows the likelihood for a range of values of K for each time step. Black denotes high likelihood and white low likelihood. Using this heat map, analysts can identify timesteps where multiple values for K are almost equally likely and where confidence in the clustering results is low.

### Electrode view

**Fig. 4:**
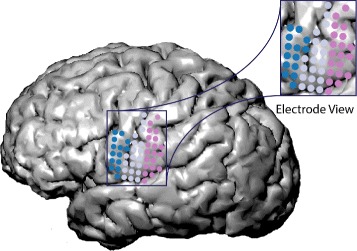
To save display space, our tool crops the electrode view to the region of the brain where electrodes are placed

**Fig. 5:**
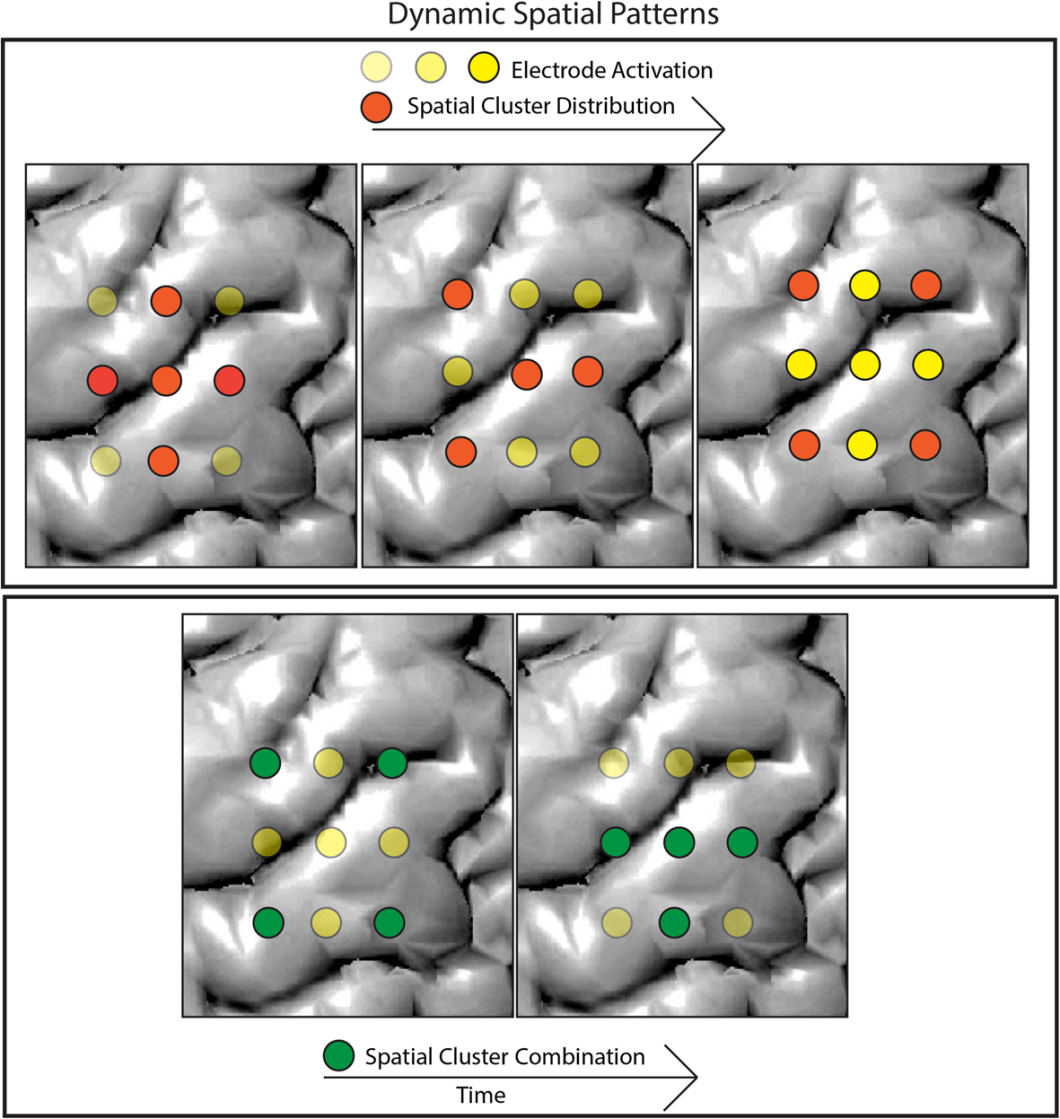
Dynamic spatial patterns captured by the electrode views. The colors of the glyphs represent clusters, and the opacity of the glyphs denote electrical activation. The *orange* cluster in the *top*, spatially distributes, while the *green* cluster in the *bottom*, spatially combines. The *yellow* cluster in the *top* activates over time

The electrode view shows (Fig. 4) cluster membership and electrical activity in a spatial context (G3) (Fig. 9 a, b), enabling the user to identify important spatial cluster evolution patterns (Q1, Q2 and Q3). These evolution patterns (Fig. 5) include 1) *spatial cluster distribution*, i.e., a cluster originally comprised of spatially adjacent electrodes splits into disjoint parts, 2) *spatial cluster combination*, i.e., a cluster consisting of disjoint regions becomes spatially coherent, and 3) *spatial activation*, i.e., the electrical activity of electrodes increases over time. The electrode view places interactive glyphs–representing electrodes–on a 2D sagittal projection of a subject-specific reconstructed brain MRI model to provide the spatial context.

To support exploration at multiple temporal scales (G2) and effective comparison of cluster characteristics—i.e., cluster membership and electrical activity of the individual electrodes—over the spatial domain, our visualization method aggregates N multiples of continuous timesteps on each electrode view. Furthermore, interactive techniques that can be applied to glyphs, make it possible for the user to define the N multiples to be aggregated on each glyph.

The aggregation techniques provide the user with a detailed overview of the underlying data while not being overwhelmed by the entire data [47]. However, aggregated views have a drawback: they can lead to cognitive information overload. Nonetheless, for the multiples we consider, we found that our visualizations are better suited for the tasks performed by our collaborating neuroscientists.

#### Glyph design

**Fig. 6:**
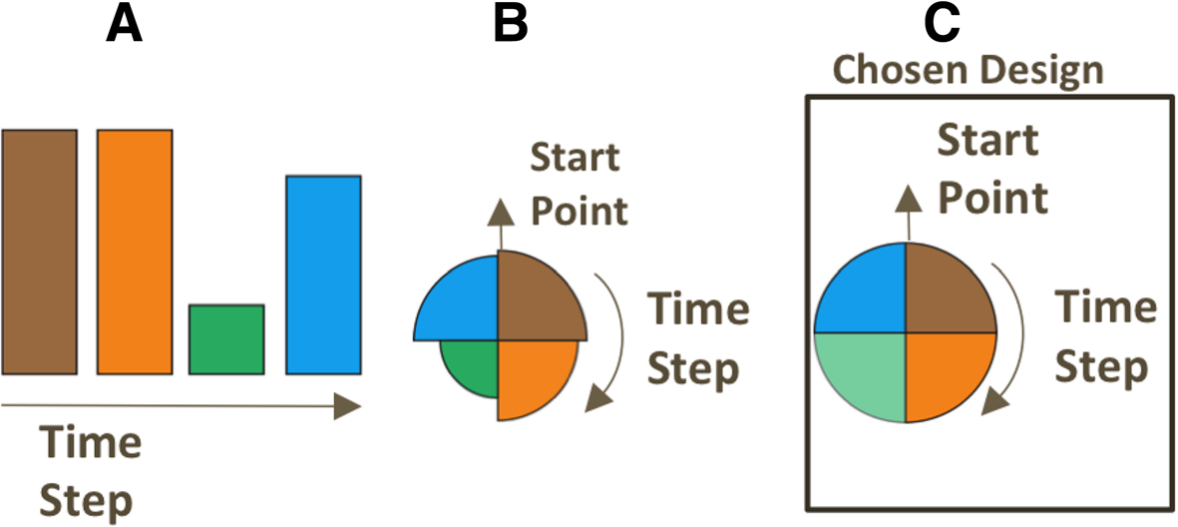
Design choices for visualizing cluster membership along with brain electrical activity. **a** The height of the individual bar correspond to the electrical activity and the color its cluster affiliation, **b** The radius corresponds to electrical activity and the color its cluster membership, **c**. The opacity of color represent the electrical activity and the color its cluster membership

All our glyph designs display *n* user-defined timesteps per individual electrode view, to save presentation space and to facilitate comparison between timesteps. We considered three visual designs for our glyphs, following some of the data aggregation guidelines specified by Elmqvist et al. [47]. The first design uses vertically stacked bar charts. Each bar represents one time step with its color indicating cluster membership and its height corresponding to electrical activity (Fig. 6 a). The second design uses a clock metaphor [32] and subdivides each glyph into *n* equal slices. Each slice—starting at the top in clockwise order—represents a time step with its color indicating cluster membership and its radius representing electrical activity (Fig. 6 b). The third design is a variation of the second design and uses slice opacity instead of radius to represent the electrical activation level (Fig. 6 c).

The first design depicts changes in cluster membership intuitively, but requires a large amount of glyph space. In the second design, the varying glyph sizes in the entire spatial layout impede the neuroscientists to assess the relative positions of the electrodes. Overall, our domain science collaborators preferred the third design where the glyph shape remains constant and only color and opacity are used to convey information. We use this design in our system and the remaining discussion in this paper is based on it.

#### Hierarchical exploration

**Fig. 7:**
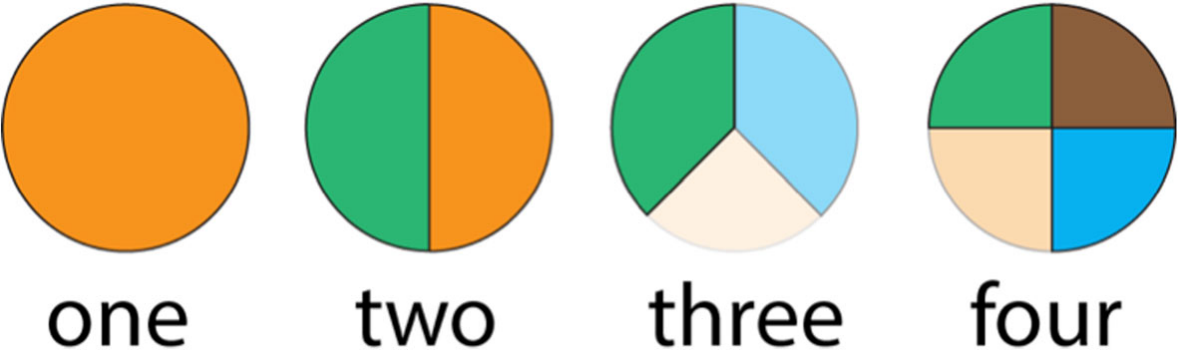
Varying the number of timesteps displayed on our glyph design

Patterns in brain activity occur at different temporal scales, where the appropriate scale may not be known a priori. To facilitate discovery of these patterns and scales, our tool controls the timesteps that can be displayed in a single electrode view (Fig. 7). At the extremes, the method either displays all timesteps in a single electrode view (low granularity at the top level) or each time step in a separate electrode view (high granularity at the bottom level). Between these extremes, different levels of aggregation are possible. Different approaches exist to search for temporal evolution patterns. *Top-down* analysis starts with all timesteps in a single view and decreases aggregation until a pattern of interest is found. A *bottom-up* approach starts analysis with each time step in a separate view and increases aggregation until a pattern is found. However, exploration may also start at *mid-level*, in situations where the user already has a notion at what temporal scale a pattern may be identified.

#### Layout

**Fig. 8:**
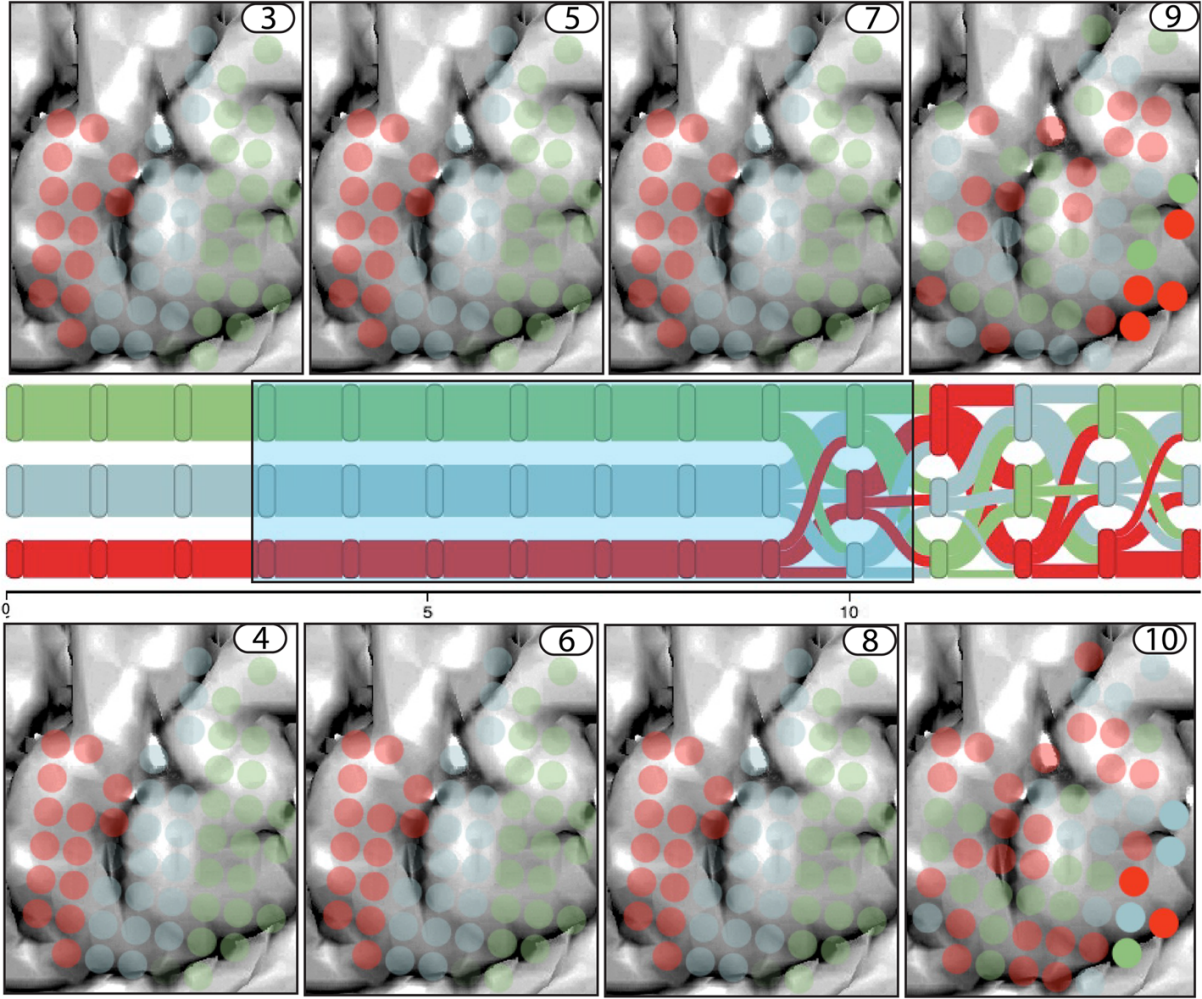
Layout for bottom-up analysis of the spatio-temporal data. The cluster evolution view is divided into equal segments and corresponds to the electrode views shown along the two horizontal axes, i.e., above (x1) and below (x2) it”

To identify low-level changes in a bottom-up based approach, several low-level electrode views need to be examined simultaneously. The system presents the views in a from-left-to-right order, synchronized with the timeline. Visual scalability is problematic as screen resolution is small relative to data resolution. Therefore, ECoG Cluster Flow utilizes a space-saving layout to achieve a tight synchronization between the spatial (electrode) view and temporal attributes (cluster evolution view) of the data. Each electrode view is ordered alternatively above and below the cluster evolution view, see Fig. 8, timesteps 3–10, to achieve the desired integration. The cluster evolution view is expanded or contracted based on the combined space allocated to all electrode views. To emphasize the granularity of the electrode view, the space assigned to each electrode view is proportional to the corresponding number of time points.

We use the formula

$$ w_{i} = w_{min}\times S_{f}, S_{f} \geq 1 $$

to define the width *w*_*i*_ of each electrode view, and

$$S_{f} = C \times \frac{W_{max}}{w_{min}\times max(N_{x_{1}},N_{x_{2}})} $$

to define the expansion factor, *S*_*f*_, for each electrode view, The variables $N_{x_{1}}$ and $N_{x_{2}}$ represent the numbers of the electrode views for the *x*_1_− and *x*_2_− axes, representing the “above” and “below” cluster evolution views, respectively, The value of *W*_*max*_ is the maximally possible horizontal screen display width, *C* is a user-specified constant, and *w*_*min*_ defines the minimal size of each electrode view.

#### Interactivity

The strength of our system is the fact that it supports fast and intuitive analysis of cluster data at interactive rates. Based on previous work [48] and the suggestions made by our collaborating neuroscientists, we selected interaction techniques satisfying the user-defined design objectives. These are the interaction principles satisfied by our system:

- **Overview first, then details on demand:** The cluster evolution view is an intuitive way for users to first obtain a simple outline of the entire dataset. Overview trends and outliers can be easily captured, enabling users to quickly determine a time interval of importance, and perform detailed exploration on the visualization.
- **Focus+context:** This technique [49] allows users to focus on small-scale evolution patterns while preserving the overall context. It utilizes smooth, animated changes to track the patterns in focus. Furthermore, the technique facilitates comparison of patterns across time-intervals over the temporal and spatial domains.
- **Highlighting and linking:** The cluster evolution view and electrode views are coordinated using brushing and linking. Users can select timesteps of interest and observe the evolution of patterns via the cluster evolution view or the electrode view. All visual elements are supported by simple tooltips providing information about the meaning of visual encodings.

## Case studies

We discuss the usefulness of our tool by considering two case studies done in collaboration with the neuroscientists and neurosurgeon on our team of co-authors. We cover two datasets in our case-studies, a synthetic and real-world seizure dataset. The real-world dataset is complicated, containing complex cluster patterns. We have therefore generated a simple, synthetic dataset with known patterns to evaluate how our visual analysis method performs in such a well-understood case. The case studies are meant to serve the purpose of demonstrating the value of our system for gaining relevant scientific insight.

### Synthetic dataset

In our setting, electrodes have (1) three known modes of electrical activity, (2) known intervals of activation patterns, and (3) known likelihood values for the K-cluster heatmap. The controlled data parameters allow us to investigate the features of the visualization outside the context of noisy brain recordings.

We create a dynamic network with 54 electrodes with activation values 0.9, 0.6, 0.3 activation values for 30 time steps, and we specify locations of the electrodes with our system. We keep the number of clusters constant for each timestep, and we define initial clusters for all electrodes in the 30 timesteps.

#### Spatio-temporal analysis:

**Fig. 9:**
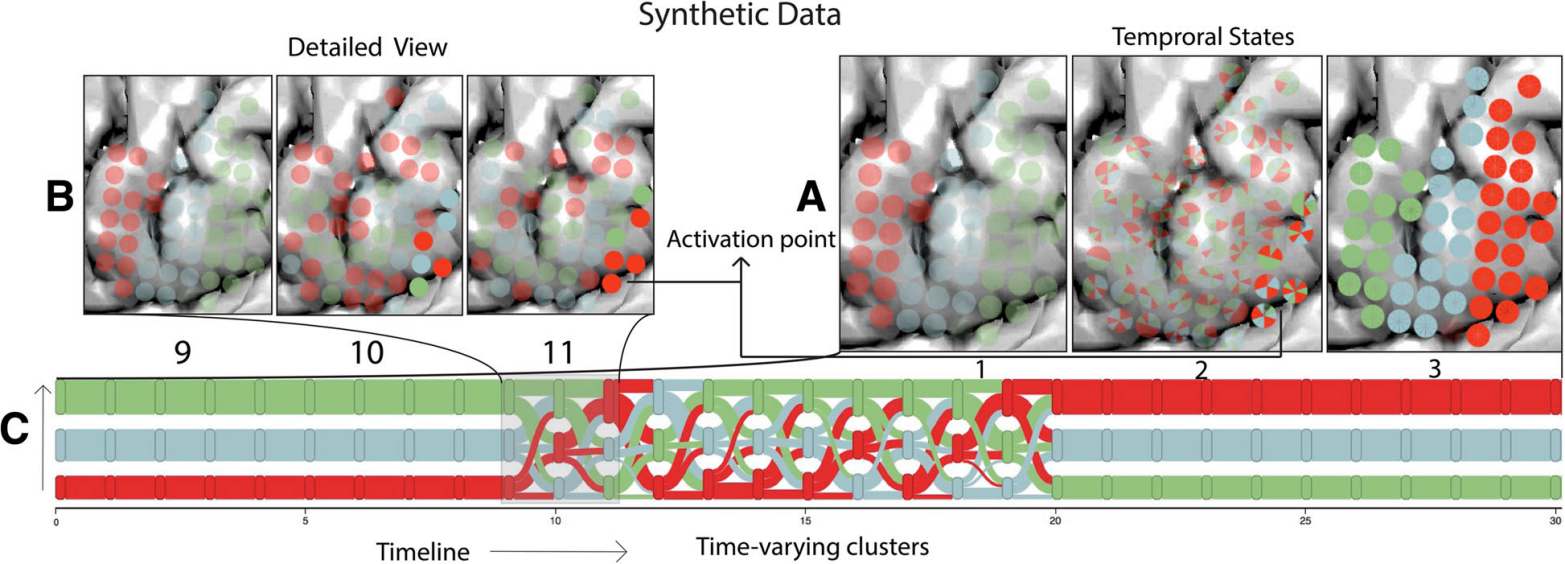
The figure shows evolution patterns underlying our generated dataset which we use to test and evaluate our approach. Color indicates the cluster configuration at each timestep and the opacities of the glyphs its electrical activity. **a** Categorizing different temporal states, i.e., unactivated (View 1), transitional (View 2), activated (View 3). **b** Evolution of the cluster assignment changes through the cluster evolution view, stable cluster configuration from timestep intervals (0–10) and (20–30), and, random unstable assignment over timestep interval (10–20). **c** Detailed analysis of a time interval selected in the cluster evolution view, activation patterns can be seen in the lower-right corner of the views for the timestep interval (10, 11)

We start our data exploration with the evolution view, looking for general evolution trends in the data. In this view, three distinct clusters (colored in red, green and blue in Fig. 9) emerge and remain stable throughout timesteps (0–9) (in Fig. 9 b). These clusters become randomly distributed at (10 to 19) to regain their stable configuration at (20 to 30).

To further examine the spatial configuration of these patterns, we employed a top-down approach exploring various temporal scales progressively to identify a consistent activation pattern (similar activation patterns in one electrode view) across all electrode views. At a granularity of ten (ten-time points in one electrode view, Fig. 9 a), persistent electrical patterns in each electrode view were found, e.g., glyphs in electrode views *one* and *three* were fully activated and deactivated, respectively. The electrode view *two*, on the other hand, showed a combination of activated and unactivated patterns.

To explore the intricate low-level activational and modular patterns that caused the temporal state change from unactivated to activated state, the granularity of the system was reduced to one (Fig. 9 b). When examining timesteps (9, 10, 11), an emergent focal activation point (annotated in Fig. 9 c) in the lower-right corner of the electrode view was evident. Further examination of subsequent timesteps revealed the progressive dominance of the red cluster over the region (Fig. 9 c, views 9, 10 and 11). In summary, our combined visual analysis approach helped us categorize temporal states and identify low-level changes and their dependencies with high-level state changes (Additional file 1).


Additional file 1: Demo of the tool with artificial dataset DemoVideo.mp4, A video demonstrating our tool in use with synthetic datasets. (MP4 34,764 kb)


### Epileptic seizure dataset

Epilepsy is a neurological condition where the normal functioning of the brain is disrupted due to sudden bursts of electrical activity emanating from a certain region of the brain, i.e., seizure-initiating foci. This disruption is characterized by changes in the brain’s modular organization over time [50]. Exploring these differences may provide insight into the genesis and development of the seizures over time [50]. The neuroscientists on our team are primarily interested in: 1) identifying the focal site of seizure genesis and its spatial progression over various stages of the seizure and 2) identifying distinct spatio-temporal evolution patterns that characterize the onset and propagation of the seizure.

**Data** The raw signal data from the ECoG electrode array was statistically analyzed to provide two spatio-temporal graph datasets using different pre-processing steps: a high-gamma dataset at a frequency ranging from 70 to 170 Hz, capturing multi-unit neuronal spiking, and a full-range dataset, averaging all frequencies captured by the recording device. We derived communities independently at each timestep using the consensus clustering algorithm [42] that automatically fits the number of clusters detected. (We note that the issue of extracting the ‘correct’communities from time-varying graphs is more of a question of the statistical data analysis algorithms used in the graph formation and community detection algorithms, than the visualization there of). With these clusters as input, our system detects and visualizes the dynamic community results in the cluster evolution and spatial view.

#### Domain expert analysis:

**Fig. 10:**
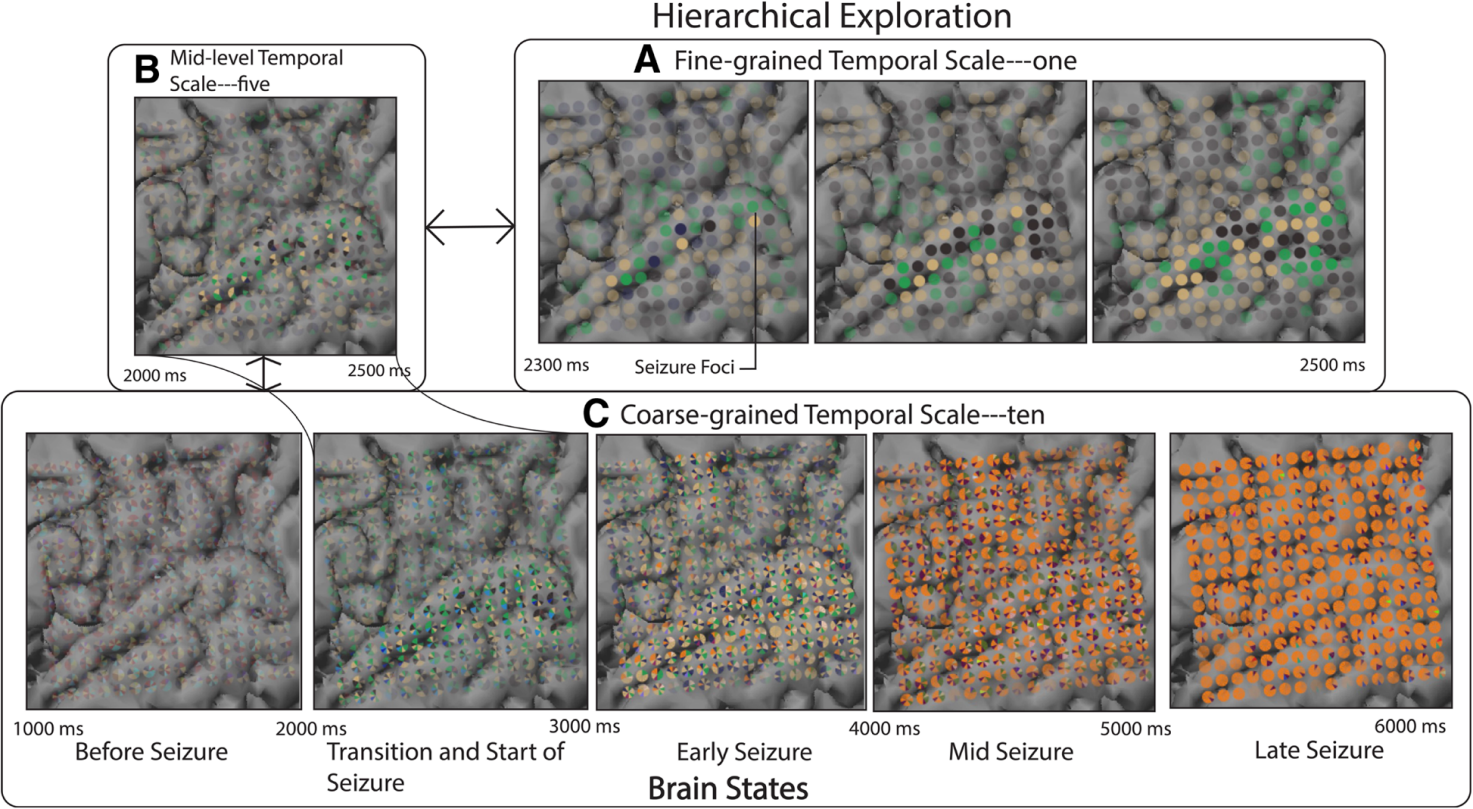
Hierarchical exploration of the seizure dataset at varying levels of granularity revealing th various brain states and associated cluster characteristics. Four major brain states are found at **c**. Upon further examination of their detailed evolution patterns in **b** and **a**, provides insight into seizure genesis and the initiation phase. The *green* cluster (in seizure initiating focii, i.e., ‘Lateral Temporal Cortex’) in **a** seems to play a prominent role in the seizure initiation phase (2200–2500 ms)
- **Detection of transitions between brain states:** Based on the gradual changes in activity patterns (2200–2600 ms in Fig. 10 a), the seizure initiation zone (2200 ms, Fig. 10 a) (cause for state change from *before-seizure* to *early-seizure*) was found. The neuroscientists stated, *“We could see activated opaque glyph patterns (Seizure Foci) over the Lateral Temporal Cortex both in Fig.* 10 a, *and in Fig.* 11 a *at 2400 ms. The opaque glyphs emerging from this point dynamically spread to other parts of the brain (3000–5000 ms in Fig.* 10 c).”

We now discuss some of the major insights obtained and details of the usage of our system by our collaborating neuroscientist and the neurosurgeon on our team:

- **Detection of brain states:** Using the bottom-up approach, around a temporal scale of ten, we found consistent progressive seizure activation patterns across all electrode views (Fig. 10 c). Based on these cluster patterns, we categorized the electrode views into four major distinct brain states, *i.e., before-seizure, early-seizure, mid-seizure, late-seizure.*

**Fig. 11:**
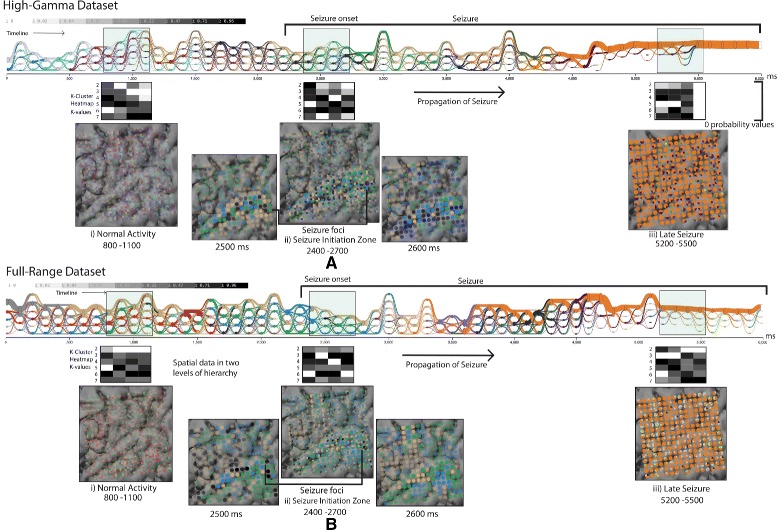
Visualizations of the two pre-processed datasets of the same raw input signals. Colors in the cluster evolution view and the electrode view indicate the time-varying communities. Three intervals are chosen for analysis to explore the modular signatures at different brain states. Significant evolution patterns at these stages are 1) normal activity—stable cluster characteristics with equally sized modules, 2) transition state—distinct activation patterns emerging from the ‘Lateral temporal cortex’, 3) late seizure state—single dominance of the orange cluster. When comparing the modular signatures between images **a** and **b**, the number of clusters in **b** seems to be larger
- **Compare and contrast the evolution patterns governing different brain states:** The signatures of cluster evolution at three distinct brain states (before-seizure, transition-state, late-seizure) were of interest to us.
  1. *800–1100 ms:* Evolution patterns here appear to be stable and organized (highly confident clusters with a clear maxima in the K-cluster heatmap), with the same number of elements in each community (Fig. 11 a, b). The spatial organization of clusters at 900 ms (Fig. 11 a) are scattered over the spatial layout and are unactivated with transparent glyphs.
  2. *2400–2700 ms:* Evolution patterns from this point onward seem to become irregular. There is a noticeable activation of certain electrodes in spatial view and reduction in number of clusters. The neuroscientist commented, *“The transitions in the cluster evolution view have a mix of irregular distribution of thin and thick links between timesteps. For example, the brown cluster in Fig.* 11 a *and* b *have comparatively less redistributions of electrode elements over time.”*
  3. *5200–5500 ms:* Concerning this period, the scientist’s comment was: *“In the cluster evolution view, a dominant orange ribbon (starting at 4000 ms) emerges with few isolated small sized communities.”* The zero likelihood in the heatmap (interval 5500–6000 ms) is caused as the algorithm produces only identity (1) values in the consensus matrix, showing no progressive change in the cumulative density plot, eventually picking the K value to be one.
- **Assess changes in community membership:** In Fig. 10 c, when evaluating the last electrode view, *there also seems to be a dominance of a single orange cluster over a significant period of time and space.*

When comparing the evolution patterns of the high-gamma and full-range pre-processed datasets (Fig. 11 a and b), the domain experts stated that, *“In general, the full-range dataset exhibits larger numbers of communities in all three sampled intervals (A,B,C). Although a single orange ribbon emerges in both datasets, it only converges clearly in the high-gamma dataset.”*

#### Domain expert review

Our collaborating neuroscientists were satisfied with the system and regarded it as intuitive and easy to use. After brief initial training, describing the visual designs and different interactions, they were able to identify regions with coherent cluster configurations for different parameter settings. The ability to switch between different views and drilling down to a level of interest was seen as very helpful by them. One comment was *“I could quickly identify general patterns of interest in the complex dataset and pick the outlier regions of interest through the powerful interactive techniques. The derived patterns are promising and reveal relevant insight into seizures, especially their genesis and overall characterization.”* Further, *“the representation of time-varying community structure and combined with the interactive multi-scale exploration approach has made possible multi-part observations for the ECoG spatiotemporal data. Without this tool, this process would have been very time-consuming and cumbersome.”*

## Discussion

Neuroscientists analyzing and visualizing spatial-temporal graph data commonly use juxtaposed small-multiples or animations of node-link diagrams with a corresponding spatial view (brain maps). We have compared our approach with these baseline visualizations methods and point out how we gain new insights through our interactive system.

**Small-multiples of brain maps:** To identify low-level community membership changes of a node over time, information in each discrete timestep and its corresponding brain map must be examined. Such static multi-view approaches make it difficult to quickly assess the stability of nodes over a local spatial region of interest. Furthermore, to identify brain states with consistent activation patterns and investigate the global cluster lifetime phenomena, nodes have to be mentally grouped and compared based on their data attributes, further increasing the cognitive load for analysis.

**Animations of brain maps:** Animations are effective in capturing low-level spatial changes of communities over a small period—a change in community membership or activation is denoted by a sudden change in color. However, the frequently changing data attributes make it challenging to infer meaningful evolution patterns over long time-scales. This complication is due to its reliance on short-term memory that requires users to remember previous static views and then manually identify and compare the relevant changes. The same holds true for identifying brain states or global cluster evolution patterns.

**Our approach:** The identification of community membership changes of a node can be achieved by choosing an appropriate level of granularity and visually analyzing it over one static view (Fig. 10 b and c). The analysis is reduced to finding the changes in the color of the consecutive slices in the glyph. Furthermore, the split-merge links in the cluster evolution view ensure that the nodes do not need to be re-identified at each timestep (small multiples) or remembered from previous timesteps (animations). Compared to these methods, interactive techniques in our approach help filter, select, zoom, pan and increase or decrease the level of granularity, help gain a thorough understanding of the significant spatio-temporal patterns in the data. Unlike the small-multiples method, our method significantly reduces the visual scalability by aggregating temporal data over the spatial electrode view.

***Findings***

In the following, we summarize our findings concerning the use of our system for exploring and analyzing ECoG data.

**Timeline-based representations enhance perception:** The tightly integrated layout mechanism, where a consistent global timeline between the cluster evolution view and the electrode views is maintained, allowed neuroscientists to correlate evolution patterns across the spatial layout and functional organization of the brain network. Furthermore, the side-by-side placement of the views on a timeline helped the scientists with the comparison of relevnt salient patterns for discontinuous timesteps.

**Bottom-up analysis versus top-down analysis:** The neuroscientists preferred using different approaches for different tasks. The bottom-up analysis approach (Fig. 10, a-b-c) was preferred over the top-down approach (Fig. 10, c-b-a) for tasks relating to Q1 and Q2. As the bottom-up exploration method generated views with increasing complexity, relationships between evolution patterns at various scales could be seen clearly. In contrast, the top-down exploration method, displaying fewer electrode views with high levels of aggregation, was cognitively overwhelming.

**Comparison of evolution patterns of multiple datasets:** In our case study, we have compared evolution patterns prevalent in two datasets. Sudden changes in cluster membership and activation data were apparent when comparing the cluster evolution view and the electrode view for the datasets. However, as both datasets have different cluster characteristics, it was difficult to determine detailed changes between the datasets. A uniform comparison criterion should ideally assign colors based on an optimization algorithm, where the same colors are used for the dynamically evolving dataset clusters that intersect. We plan to devise a method to achieve this in the future.

Scalability is a main design consideration that we took into account when designing ECoG Cluster Flow. Hierarchical representation/visualization and data aggregation methods are primary technqiues that we employ to explore the high-resolution spatio-temporal data. We have visualized up to 64 timesteps and 256 electrodes. A challenge will be to scale the system’s real-time capability to much larger datasets. Our goal is to support interactive, real-time visual analysis for datasets with more than 500 timesteps. Further, to keep the amount of visual representation at a level that can be comprehended by a human, we will devise and employ data reduction and abstraction techniques to simplify data visualization.

## Availability and requirements

ECoG Cluster Flow is implemented in Python as a standalone application in conjunction with D3.js and QPainter. ECoG Cluster Flow will be available as a free open-source software, along with documentation and a demo video.

## Conclusions

We have presented ECoG ClusterFlow, a hierarchical multi-scale approach for visualizing spatial and functional cluster evolution patterns. Our approach has allowed neuroscientists to investigate the major cluster evolution patterns over space and time. Through our approach, it is possible to examine whether the major evolution events were a result of noise or a sudden change in functional or spatial properties of the network. Furthermore, we have discussed major neuroscience-driven data analysis tasks and design choices that led to the entire design of the system (done in close collaboration with neuroscientists) that helped gain insights for the spatial cluster evolution data.

The visual analysis approach greatly supports the comprehension of salient spatio-temporal evolution patterns and provides insight into the life-span of brain states and the clustering stability of the classification algorithm.

We plan to perform an evaluation concerning the usefulness of spatio-temporal clustering, i.e., a clustering algorithm that takes both the spatial as well as the temporal attributes into account. When utilizing both these attributes, interesting research questions arise, such as, *“is the resulting visualization an accurate representation of the data? How can we consistently compare such types of visualizations with a different dataset?”*.

## Abbreviation

ECoG: Electrocorticography
